# AOP Key Event Relationship report: Linking decreased retinoic acid levels with disrupted meiosis in developing oocytes

**DOI:** 10.1016/j.crtox.2022.100069

**Published:** 2022-03-18

**Authors:** Monica Kam Draskau, Anne-Sofie Ravn Ballegaard, Louise Ramhøj, Josephine Bowles, Terje Svingen, Cassy M. Spiller

**Affiliations:** a National Food Institute, Technical University of Denmark, Kgs. Lyngby DK-2800, Denmark; b School of Biomedical Sciences, The University of Queensland, Brisbane, QLD 4072, Australia

**Keywords:** Adverse Outcome Pathway, Key event relationship, Retinoic acid, Ovary, Meiosis, Germ cells, Infertility

## Abstract

The Adverse Outcome Pathway (AOP) concept is an emerging tool in regulatory toxicology that uses simplified descriptions to show cause-effect relationships between stressors and toxicity outcomes in intact organisms. The AOP structure is a modular framework, with Key Event Relationships (KERs) representing the unit of causal relationship based on existing knowledge, describing the connection between two Key Events. Because KERs are the only unit to support inference it has been argued recently that KERs should be recognized as the core building blocks of knowledge assembly within the AOP-Knowledge Base. Herein, we present a first case to support this proposal and provide a full description of a KER linking decreased all-trans retinoic acid (atRA) levels in developing ovaries with disrupted meiotic entry of oogonia. We outline the evidence to support a role for atRA in inducing meiosis in oogonia across mammals; this is important because elements of the RA synthesis/degradation pathway are recognized targets for numerous environmental chemicals. The KER we describe will be used to support an intended AOP linking inhibition of the atRA producing ALDH1A enzymes with reduced fertility in women.

## Highlights

• The first case study to develop and publish an individual KER as a stand-alone unit of information under the AOP framework overseen by the OECD.
• Full description of a KER linking decreased all-trans retinoic acid (atRA) levels in developing ovaries with disrupted meiotic entry of oogonia.
• KER described is associated with an intended AOP linking inhibition of the atRA producing ALDH1A enzymes with reduced fertility in women.

## Graphical abstract

**Figure:**
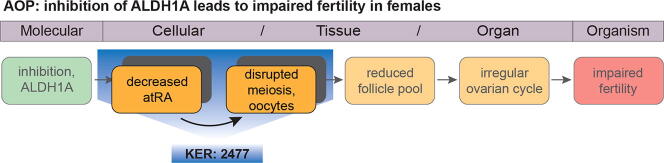

## Pretext

The Adverse Outcome Pathway (AOP) framework has emerged as a promising tool to aid in toxicological risk assessment across disciplines. Its intention is to provide readily available descriptions of causal chains of events linking an interaction with a stressor, a so-called Molecular Initiating Event (MIE), with an adverse outcome (AO) in an intact organism. Although simple in principle, this approach has proven challenging in practice, as the practical development of robust AOPs comprising all required information is very resource demanding. In addition, individual AOPs often span several specialized fields of expertise; this provides a challenge not just for the AOP developers, but also for acquiring suitable external reviewers. To address these challenges, we recently proposed that smaller units of information within the AOP framework should be expertly reviewed using the standard scientific journal peer-review processes (Svingen et al., 2021). Although the success of this new review method will likely depend on the complexity of the AOP and also the strength of evidence for a given Key Event Relationship (KER) (discussed in Svingen et al., 2021), we expect that in the vast majority of cases, adoption of this as a practice will allow AOP development and implementation to be accelerated.

Since KERs describe biologically plausible links between upstream and downstream events, we proposed to use the KER as the core unit of individually recognized building blocks. This report provides a case example of this approach, where we link reduced levels of all-trans retinoic acid (atRA) in the fetal ovary with disrupted meiosis in the oocytes. The rationale for selecting this KER is that it provides the central unit of knowledge linking disrupted retinoic acid signaling during development with compromised fertility later in life, as well as being ‘emerging evidence’ in the sense that the knowledge is not considered canonical. Thus, we provide available evidence for showing a biologically plausible link between two canonical Key Events (KEs).

## Introduction

The primordial germ cells, which eventually give rise to eggs or sperm, are initially bipotential; they later adopt their sexual fate in response to their local somatic environment during fetal life. In the fetal ovary, the germ cells enter meiotic prophase I shortly after the ovaries have differentiated during mid-gestation and progress through leptotene, zygotene and pachytene stages before arresting in late diplotene stage before birth (Spiller et al., 2017). The pre-meiotic and early meiosis state is marked by expression of *Stra8* (*Stimulated by retinoic acid gene 8*), *Meiosin* (meiosis initiator, previously GM4969) and *Rec8* (Bowles et al., 2006, Ishiguro et al., 2020, Koubova et al., 2014). Other later markers of meiosis include those involved in assembling the meiotic machinery and the formation of double strand breaks, including SYCP3 (synaptonemal complex protein 3) and γH2AX (H2A.X variant histone) (Baltus et al., 2006). Of these markers, STRA8 is arguably of greatest value as it is cleanly switched from OFF to ON at the point of meiotic commitment and because its expression is highly sensitive to atRA availability. STRA8 is crucial for meiosis because *Stra8*-null mice of both sexes are infertile due to failed entry and progression within meiotic prophase (Anderson et al., 2008, Baltus et al., 2006, Mark et al., 2008).

There is now ample evidence that atRA is a central signaling molecule for inducing *Stra8* expression, and therefore meiosis, during fetal life in mammalian oocytes (reviewed by Griswold et al., 2012; Jorgensen and Rajpert-De Meyts, 2014, Spiller et al., 2017). Perhaps most compellingly, with respect to proof of an *in vivo* requirement for atRA, is the finding that when vitamin A (retinol, the precursor to atRA) is depleted from the diet in pregnant rats (VAD; vitamin A deficient), germ cells in the female embryos do not enter meiosis but remain undifferentiated (Li & Clagett-Dame, 2009). Although there has been some debate as to whether atRA is essential to ensure germ cells of the fetal ovary enter meiosis, it is clear that atRA signalling is necessary for normal levels of *Stra8* expression (Chassot et al., 2020, Feng et al., 2021, Kumar et al., 2011; Spiller, 2022; Vernet et al., 2020).

There seems to be conservation between rodent and human in terms of the requirement for atRA to induce the pre-meiotic factor STRA8; however, the source of atRA during gonadal development appears to differ between species (Childs et al., 2011). In mice, the adjacent mesonephros, which expresses two ALDH1A (also known as RALDH) enzymes able to catalyse the final step in atRA production, ALDH1A2 and ALDH1A3, is likely the main source of atRA at early developmental stages (Bowles et al., 2018, Bowles et al., 2006, Niederreither et al., 1999). There is also the capacity for lower concentrations of atRA to be produced within the mouse ovary itself, with the local expression of ALDH1A1 (Bowles et al., 2016, Mu et al., 2013). In humans, ALDH1A enzymes (ALDH1A, −1B and −1C) are expressed in both testes and ovaries of the developing fetus, which suggest a capacity for *de novo* atRA synthesis within the gonads (Childs et al., 2011, Jorgensen and Rajpert-De Meyts, 2014, Le Bouffant et al., 2010) as is also the case in rabbits (Diaz-Hernandez et al., 2019). In humans, a peak of ALDH1A1 expression at the onset of meiosis has been reported (Le Bouffant et al., 2010), which suggests that meiotic onset is dependent on timely provision of atRA.

Because atRA synthesis is a known target for certain environmental chemicals, for instance the thiocarbamate herbicides pebulate, vernolate, butylate and tri-allate (Quistad et al., 1994), cyanamide (Nagasawa et al., 1990, Shirota et al., 1987) and the isoflavone daidzin (Lowe et al., 2008), the retinoid signaling pathway should be considered in chemical safety assessment and regulation. Indeed, this notion was proposed by the OECD almost a decade ago in a Detailed Review Paper (OECD, 2012). A recently published ‘Detailed Review Paper on the Retinoid Signaling Pathway’ (supported by the European Commission and OECD) included an annex focusing on both male and female reproduction (OECD, 2021). But despite these efforts there are still no AOPs for disrupted RA signaling in the AOP-Knowledge Base (AOP-KB) even though several putative AOPs involving retinoid disruption have been suggested (Draskau et al., 2020, Johansson et al., 2020, Knudsen et al., 2021, Tonk et al., 2015). We currently have under development AOP 398 ‘Inhibition of ALDH1A (RALDH) causing reduced all-trans retinoic acid levels leading to impaired fertility’ and here describe a relevant KER that links reduced atRA levels with disrupted meiosis in fetal oocytes.

## Linking KER 2477 to an AOP

The unit of knowledge presented in this report (a KER linking decreased atRA levels in the fetal ovary and disrupted meiotic onset) is part of a proposed AOP linking inhibition of ALDH1A as a MIE with decreased fertility in females as an AO. This AOP is available in AOP-wiki (https://aopwiki.org/aops/398) along with the descriptions of the relevant KEs and KERs, including that described in the following. The sections where the text is identical, at the time of peer-review, are denoted by (*[access date]) in the overarching heading.

**Fig. 1:**
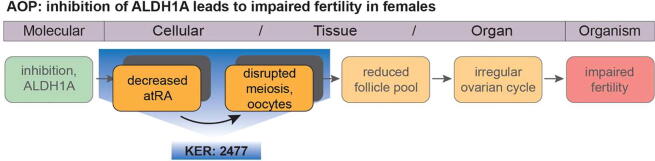
**Graphical representation of AOP 398.** The AOP proposes a causal link between inhibition of ALDH1A action during ovary development and impaired fertility in females at reproductive age. A key step is a decrease in all-trans retinoic acid (atRA) in the fetal ovaries at the time when germ cells (oocytes) normally enter meiosis. As atRA is a key meiosis-inducing factor, decreased levels of atRA prevents or impairs a critical step in oocyte development (meiosis onset); subsequently this negatively affects oocyte development, the size of the oocyte pool (ovarian reserve) and ultimately, female fertility. Key Event Relationship (KER) 2477 describes the biologically-plausible link between decreased atRA in fetal ovaries and disrupted meiotic onset in oocytes.

Figure 1 gives a graphical representation of the AOP and shows the step in the pathway that is relevant to the KER presented here.

## Literature search strategy

To provide an overview of available literature a search was performed in PubMed. The search was conducted with the terms “retinoic acid AND meiosis AND (ovary OR oocytes)” (search performed 23 June 2021). The search resulted in 120 hits (full list in **Table S1**). The list was sorted based on title and abstract according to the relevance to the KER, which included further limiting papers to the 37 that addressed 1) fetal development and 2) ovarian development, specifically (rather than testis development).

## Key Events flanking KER 2477

In cases where KEs have already been developed and reviewed (or endorsed), the KE descriptions need to be accessed via AOP-wiki through their unique IDs. In this report, the two flanking KEs have been developed alongside the KER, so they are included herein for initial review. Any future updates or amendments to the KEs will be freely available at AOP.wiki.org.

### KE 1881: Decreased, atRA levels (*accessed February 23, 2022)

#### Key Event description

All-trans retinoic acid (atRA) is the active form of vitamin A/all-trans retinol and is involved in regulating a large number of developmental processes (Bushue and Wan, 2010, Ghyselinck and Duester, 2019). Although 9-cis RA and 13-cis RA are other metabolic derivatives of vitamin A, atRA is generally considered the primary active metabolite during development, mainly acting as a short-range paracrine signaling molecule (Cunningham & Duester, 2015). atRA exerts dose-dependent effects on morphogenesis, so disruption to atRA concentrations during development can lead to malformations in numerous tissues and organs. During development the spatiotemporal regulation of atRA concentrations in target tissues is tightly controlled by a balance of synthesis and degradation enzymes (Kedishvili, 2013).

Cellular atRA synthesis starts by oxidation of vitamin A to retinaldehyde (RAL) by retinol dehydrogenase-10 (RDH10). RAL is then irreversibly converted to atRA by RAL dehydrogenases (ALDH1A1, ALD1A2, or ALDH1A3). To maintain appropriate retinoid levels in tissues, RAL can be converted back to retinol by enzymatic reactions; further retinoid levels can be controlled by enzymatic degradation of atRA by the cytochrome P450 enzymes CYP26A1, CYP26B1, or CYP26C1, which are differentially expressed throughout the mammalian body (Isoherranen and Zhong, 2019, Shimozono et al., 2013). Inhibition/disruption of any of the enzymes of the atRA synthesis pathway, or increased expression of the atRA degradation enzymes can lead to decreased concentrations of atRA in target cells (Kedishvili, 2013).

The atRA functions as a ligand for the nuclear retinoic acid receptors (RARs), which form heterodimers with the retinoid X receptors (RXRs); the atRA:RAR:RXR complex then binds to retinoic acid response elements (RAREs) upstream of target genes, leading to activation or repression of gene expression in target cells (Chambon, 1996, le Maire et al., 2019). The type and number of RAR/RXRs differ between evolutionary distant animals, but functionally they are all involved in the regulation of development (Gutierrez-Mazariegos et al., 2014).

#### General notes on taxonomy and domain of application

The specifics of these sub-categories, presented as tables, can be assessed at AOP-wiki under KE ID no. 1881. In brief, the taxonomic applicability includes vertebrates most broadly, but with evidence originating mostly from zebrafish, mouse, rat and human. The domain of applicability includes both sexes, across developmental stages and in numerous tissues and organs throughout the body plan. The retinoid signaling system is highly conserved across animal species (Bushue and Wan, 2010, Rhinn and Dolle, 2012).

#### How the KE can be measured or detected

Direct measurements of atRA in serum (humans, animals) can be performed by various chromatographic methods (Gundersen, 2006), including high performance liquid chromatography (HPLC) or liquid chromatography-tandem mass spectrometry (LC-MS) (Morgenstern et al., 2021).

Indirect measurements in cells and animal models can be performed with reporter assays utilizing RAR-RXR-RARE or RXR-RXR-RARE promoter elements, which are activated by atRA, driving expression of reporter proteins. These reporter assays can detect the presence of atRA in tissues in a semi-quantitative manner. Examples include reporter mouse lines (Carlsen et al., 2021, Rossant et al., 1991, Solomin et al., 1998), reporter cell lines (Wagner et al., 1992) and transient transfection of constructs for *in vitro* cell-based assays (Chassot et al., 2020).

#### Evidence for perturbation by stressor

WIN 18446

WIN 18446 is an ALDH1A2 inhibitor, inhibits RA biosynthesis resulting in reduced atRA in mice: e.g. around 50% reduction in liver and 90% reduction in testes (Arnold et al., 2015).

Ethanol

Ethanol is also catalyzed by the enzyme alcohol dehydrogenase (ADH) and is known to compete with retinol for ADH-catalyzed oxidation for production of retinoic acid: ethanol treatment in embryonic day 7.5 mouse embryos reduced the amount of atRA produced, detected using transgenic reporter cell lines (Deltour et al., 1996).

Diethylaminobenzaldehyde

Diethylaminobenzaldehyde (DEAB), an ALDH inhibitor: treatment of zebrafish embryos with DEAB reduced atRA signalling, detected using a transgenic reporter zebrafish model (Le et al., 2012).

### KE 1882: Disrupted meiosis, oocytes (*accessed February 23, 2022)

#### Key Event description

Oocyte meiosis

Oogonia, the female germ cells, are the precursors for the female oocytes. Primary oocytes are formed in the ovaries during fetal development when oogonia enter into prophase I of meiosis; meiotic entry initiates at around embryonic (E) day 13.5 in mice, E15.5 in rats, and gestational week 10–12 in humans. The entry into meiosis is driven by expression of the key genes *Stra8, Meiosin* and *Rec8* and is followed by expression of meiotic proteins including SYCP3 and γH2AX (Baltus et al., 2006, Bowles et al., 2006, Ishiguro et al., 2020, Kojima et al., 2019, Koubova et al., 2014, Spiller et al., 2017). The crucial role for *Stra8* in meiotic entry is conserved from mice to humans (Childs et al., 2011).

Disrupted meiotic entry as a Key Event

The initiation of meiosis during fetal life is critical for maintenance of the oocytes throughout development and, eventually, for establishing the oocyte pool, or ‘oocyte reserve’ at birth. Without timely fetal entry into meiosis, the oogonia are depleted, as evidenced in *Stra8*-null mice (Baltus et al., 2006). The *Stra8*-null female mice are infertile and display abnormally small ovaries that are devoid of oocytes. For *Stra8* to be expressed and, therefore, for meiosis to initiate, the oogonia require direct stimulation by atRA as evidenced in mice (Bowles et al., 2016, Bowles et al., 2006, Feng et al., 2021, Koubova et al., 2006, Spiller et al., 2017, Teletin et al., 2017), and humans (Childs et al., 2011, Le Bouffant et al., 2010).

#### General notes on taxonomy and domain of application

The specifics of these sub-categories, presented as tables, can be assessed at AOP-wiki under KE ID 1882. In brief, the taxonomic applicability includes mouse, rat and humans (and likely many more mammalian species). The domain of applicability includes only the female sex and relates to fetal life stages in mouse, rat and human. It is established that fetal oogonia need to enter meiotic prophase I to establish the oocyte pool and this process in conserved between mice, rats, humans and more.

#### How the KE can be measured or assessed

There are no OECD-validated assays for measuring meiotic inhibition.

The expression of meiotic factors, such as STRA8, SYCP3, γH2AX, can be assessed at mRNA and/or protein levels and levels measured using primers/probes and antibodies that are commercially available.

Indirect measurements in animal models can be performed using the *Stra8* promoter element driving expression of reporter protein GFP (Feng et al., 2021). This reporter assay can detect the presence (GFP) or absence (GFP negative) of *Stra8* promoter activation in a semi-quantitative manner.

#### Evidence for perturbation by stressor

Stressors that have been shown to disrupt meiotic entry of female germ cells include:

Acetaminophen

Paracetamol (acetaminophen) exposure (350 mg/kg bw/day between E13.5 and E21.5) delayed meiotic entry in rat fetal ovaries, seen with delayed *Stra8* expression (Dean et al., 2016). In mice, paracetamol exposure (50 and 150 mg/kg bw/day, from E7 to birth) did not affect *Stra8* expression, yet oocyte numbers were decreased (Holm et al., 2016).

Indomethacin

Indomethacin exposure delays meiotic entry in rat fetal ovaries, seen with delayed *Stra8* expression (Dean et al., 2016).

Bis(2-ethylhexyl) phthalate

Diethyl hexyl phthalate (DEHP) exposure at a dose of 40 µg kg^−1^ from E0.5 to E18.5, caused delayed meiosis of oocytes, evident by delayed *Stra8* expression and meiotic progression determined by SYCP3 staining of chromosome spreads (Zhang et al., 2015). An *in vitro* model reported the same delay to meiosis when E12.5 ovaries were cultured in 10 µM and 100 µM concentrations of DEHP (Liu et al., 2017).

Bisphenol A

Bisphenol A (BPA) exposure may delay entry into meiotic prophase I in mice, potentially through reduced *Stra8* expression (Zhang et al., 2012). This effect from BPA exposure was not seen in a second mouse study (Lawson et al., 2011), nor in human ovary explant cultures (Brieno-Enriquez et al., 2012). As such, it remains uncertain if BPA can prevent oocytes from entering meiosis.

## KER 2477: Decreased atRA levels lead to disrupted meiosis in oocytes (*accessed February 23, 2022)

### KER description

All-trans retinoic acid (atRA) is the active metabolite of vitamin A and is involved in regulating a large number of developmental processes (Bushue and Wan, 2010, Ghyselinck and Duester, 2019). atRA is produced in spatial and temporal gradients, and these patterns are maintained by regulated expression of the synthesis and degradation enzymes of the atRA pathway (Kedishvili, 2013).

The presence of atRA in the fetal ovaries induces germ cells to enter meiosis (Spiller et al., 2017). The initiation of meiosis at this time during fetal life is critical for maintenance of the germ line throughout development and establishment of the oocyte pool at birth. If atRA is not present at the correct time and at sufficient concentration, meiotic initiation is either delayed or prevented from occurring, ultimately disrupting germ cell development.

### Evidence supporting this KER

#### Evidence summary

The majority of evidence for this KER comes from rodent studies. In pregnant rats, depletion of vitamin A, the precursor of atRA, leads to an inability of ovarian germ cells to initiate meiosis (Li & Clagett-Dame, 2009). Further studies in mice have produced strong evidence that atRA acts as a meiosis-inducing factor in oogonia of the ovaries, although there are some conflicting data depending on which techniques are used (Griswold et al., 2012; Spiller, 2022). Evidence for the same mechanisms in human fetal ovaries is less substantiated and there may be species differences, particularly the manner in which atRA is made available (reviewed by Jorgensen & Rajpert-De Meyts, 2014). In humans, evidence to support this KER comes from studies using explanted ovary culture.

#### Biological plausibility

In mammalian germ cells, the initiation and progression of meiosis is critically dependent on the expression of Stimulated by retinoic acid gene 8 (*Stra8*). In mice, deleting *Stra8* leads to infertility in both males and females due to meiotic failure (Anderson et al., 2008, Baltus et al., 2006, Mark et al., 2008). What regulates the temporal expression of *Stra8,* and other factors (such as *Meiosin* and *Rec8*) in the germ cells is not completely clear, but there is strong evidence to support an important and direct role for atRA (Bowles et al., 2006, Feng et al., 2021, Griswold et al., 2012, Koubova et al., 2014, Li and Clagett-Dame, 2009, Soh et al., 2015).

In the fetal mouse ovary, entry into meiosis, preceded by *Stra8* expression, occurs in an overlapping anterior-to-posterior wave from embryonic day (E) 12.5 (Bowles et al., 2006, Menke et al., 2003). *Stra8* is also expressed in rat oogonia at comparative developmental stages to the mouse (Liu et al., 2020). atRA can similarly upregulate *Stra8 in vitro*, but this is restricted to pluripotent cell lines (Feng et al., 2021, Oulad-Abdelghani et al., 1996, Wang et al., 2016). Culture of mouse skin-derived stem cells with atRA stimulates the formation of functioning gametes and improves oogonia-like cells entry into meiosis (Dyce et al., 2018, Miyauchi et al., 2017). *Stra8* expression cannot be induced by atRA in non-pluripotent cell lines, nor in somatic cells *in vivo* (Feng et al., 2021).

Exposure of pre-meiotic tammar (marsupial) ovaries to atRA induces *Stra8* expression and oogonial meiotic entry (Hickford et al., 2017). Culturing fetal mouse ovaries in the presence of atRA increases the number of meiotic oocytes (Livera et al., 2000) and the same phenomenon is observed in cultured human fetal ovaries (Jorgensen et al., 2015).

In mouse ovaries lacking the atRA synthesizing enzyme ALDH1A1, the onset of germ cell meiosis is delayed (Bowles et al., 2016). This supports a previous study showing that atRA derived from the ovary (rather than mesonephros) is sufficient to initiate meiosis in mice (Mu et al., 2013). In humans, the local synthesis of atRA by ALDH1A enzymes within the ovary may also be involved in meiotic regulation (Childs et al., 2011, Le Bouffant et al., 2010). In two recent studies looking at mouse ovaries lacking all known atRA synthesizing enzymes (Chassot et al., 2020) or RA receptors (Vernet et al., 2020), expression of *Stra8* was delayed, albeit some meiosis was still observed in these mice.

#### Empirical support for linkage

For animal model supporting data, see Table 1. For *in vitro* / *ex vivo* supporting data, see Table 2.

**Table 1:** Animal models.

Model	Relevant observations	Reference
Vitamin A deficient (VAD) rats	Oocytes fail to enter meiosis in ovaries of VAD rats due to atRA deficiency. Meiotic entry measured by SYCP3 expression was detected in 10% and 30% of germ cells in rats fed severely deficient (1.5μg of atRA per gram of diet) and moderately deficient (12μg of atRA per gram of diet) atRA diets, respectively, whilst controls had 70% of germ cells enter meiosis. The expression of the atRA-responsive gene, *Stra8*, was reduced by approximately 90% and 50% in the severely and moderately atRA-deficient ovaries, respectively, compared with the atRA-sufficent controls.	(Li & Clagett-Dame, 2009)

**Table 2:** *In vitro*/*ex vivo* evidence.

Study type	Species	Compound	Effect Dose	Duration	Results	Reference
Fetal ovaries in culture	Mouse	WIN 18,446 (ALDH1A2 inhibitor)	2 µM	3–12 d	Reduced *Stra8* expression and germ cell loss	(Rosario et al., 2020)
Fetal ovaries in culture	Mouse	BMS-189453 (RAR antagonist)	1 µM	3 d	Reduced STRA8-positve germ cells without overall oocyte loss	(Minkina et al., 2017)
Embryonic stem cells	Mouse	atRA	100 nM		Activates meiosis-related gene network	(Aoki & Takada, 2012)
Embryonic stem cells	Mouse	BMS-493 (RAR antagonist)	10 µM		Inhibition of expression of meiosis-related genes	(Aoki & Takada, 2012)
Naked oocytes, matured	Mouse	atRA	2 µM	24 h	Culture in presence of atRA increased meiosis resumption and formation of metaphase II oocytes	(Tahaei et al., 2011)
fetal ovaries in culture	Human	atRA	1 µM	1–3 d	atRA strongly promoted initiation of germ cell meiosis	(Le Bouffant et al., 2010)
fetal ovaries in culture	Human	BMS-189453 (RAR antagonist)	10 µM	14 d	Partial inhibition of meiotic entry of germ cells	(Le Bouffant et al., 2010)
fetal ovaries in culture	Human	Citral	55 µM	14 d	Partial inhibition of meiotic entry of germ cells by inhibiting RA synthesizing enzymes	(Le Bouffant et al., 2010)
Fetal ovaries in culture	Mouse	AGN193109 (RAR antagonist)	5 µM	48 h or 72 h	Meiotic program inhibited	(Bowles et al., 2006)
Fetal ovaries in culture	Mouse	BMS-204493 (RAR antagonist)	5 µM	2 d	*Stra8* expression not upregulated in germ cells, failed initiation of meiosis	(Koubova et al., 2006)
Fetal ovaries in culture	Mouse	atRA	1 µM		Acceleration of germ cells into meiosis, reduction in total number of germ cells	(Livera et al., 2000)
Fetal ovaries in culture	Mouse	CD0336 (RARα agonist)	1 nM		Acceleration of germ cells into meiosis, reduction in total number of germ cells	(Livera et al., 2000)
Naked oocytes, matured	Camel	atRA	20 µM	24 h	Stimulates meiosis and promotes oocyte viability	(Saadeldin et al., 2019)
Fetal ovaries in culture	Chicken	atRA	1 µM		Stimulates meiotic initiation	(Yu et al., 2013)

#### Uncertainties or inconsistencies

Mouse deletion models for the atRA synthesis enzymes *Aldh1a1*, *Aldh1a2* and *Aldh1a3* showed decreased expression of *Stra8* in double (*Aldh1a2/3*) and triple (*Aldh1a1/2/3*) knockouts, although ultimately some germ cells were observed undergoing meiosis in these ovaries, suggesting that atRA is not essential for meiotic onset or progression (Chassot et al., 2020, Kumar et al., 2011). Similarly, transgenic mice lacking the three atRA nuclear receptors (RAR-α, -β, -γ) showed reduced levels of *Stra8*, although ultimately some germ cells were observed undergoing meiosis and were capable of producing live offspring (Vernet et al., 2020). Whether or not these models led to impaired fertility (such as sub-fertility) has not been elucidated and the size of their oocyte pools were not determined. In addition, the completeness of the genetic deletions in these models is not clear (discussed in Spiller, 2022).

Gain of function mouse ovary models for CYP26A1 and CYP26B1 show that CYP26B1 can prevent oocytes from entering meiosis (as assessed by failure to induce *Stra8* expression), whereas CYP26A1 does not have the same effect despite being a potent atRA degrading enzyme. This suggests that CYP26B1 works by additional mechanism(s) other than RA degradation (Bellutti et al., 2019).

### Quantitative understanding of the KER

#### Quantitative understanding

The quantitative knowledge pertaining to this KER is very limited as little is known about 1) the levels of endogenous atRA produced in the ovaries in different mammals and 2) the levels of atRA required to achieve meiotic initiation.

#### Response-response relationship

*In vitro* and *ex vivo*, it has been conclusively shown that low levels (as low as 1 µM) of exogenous atRA can induce germ cells to enter meiosis in mice (Bowles et al., 2010) and rats (Livera et al., 2000) and, similarly, that it is necessary to achieve meiosis in *in vitro*-derived oocytes via primordial germ cells (PGCs)/PGC-like cells (PGCLCs) (Miyauchi et al., 2017). Yet, its exact role *in vivo* is under debate.

Whilst the relative levels of endogenous atRA produced by the ovary (for any species) remains unknown, similarly, the quantitative relationship between atRA levels and induction of meiosis also remains unclear. As such, the quantitative understanding of how much atRA needs to be reduced to prevent germ cells to enter meiosis *in vivo* is rated low.

#### Time scale

The time-scale for this KER is relatively short, limited to just a couple of days in mice, and several weeks in humans. The induction of meiosis occurs shortly after the germ cells have colonized the ovary and occurs asynchronously (Bullejos & Koopman, 2004) (in mice this begins at E13.5 and is completed for all germ cells two days later at E15.5). Proliferation is halted and cells progress through leptonema, zygonema, pachynema, and arrest in diplonema of prophase I prior to birth (Zamboni, 1986). Time and duration of progression through prophase I varies between species, with rats the shortest duration of only 1–2 days, with other mammals such as pigs, cows, monkeys and humans lasting months (Peters, 1970).

The rat model of vitamin A deficiency (VAD) revealed severe defects to meiosis induction when dietary Vitamin A was restricted at E10.5, which is just 3 days prior to normal meiotic induction (Li & Clagett-Dame, 2009). Shorter timeframes have not been assessed to date, nor has rescue of VAD during later embryonic time-points been attempted.

#### Known modulation factors

No modulating factors are currently known to alter the quantitative relationship between the two KEs.

#### Known feedforward/feedback loops influencing this KER

During development, RA homeostasis is regulated by feedback loops, as both too much and too little RA can have deleterious effects on the embryo or fetus. The availability of atRA is regulated locally by maintaining a balance between synthesis (ALDH1A enzymes) and metabolism (CYP26 enzymes) (Kedishvili, 2013, Niederreither and Dolle, 2008, Roberts, 2020, Teletin et al., 2017).

The expression of *Aldh1a2* and *Cyp26a1* can act as part of a negative feedback loop in response to changes in RA levels. Exogenous atRA suppresses expression of *Aldh1a2* (Niederreither et al., 1997) whereas blocking atRA signalling increases expression of *Aldh1a2*. Although *Cyp26a1* expression does not require atRA, addition of atRA greatly increases the expression of *Cyp26a1*, and conversely, reduced levels of atRA reduces *Cyp26a1* expression (de Roos et al., 1999, Hollemann et al., 1998, Ross and Zolfaghari, 2011, Sirbu et al., 2005). Negative feedback loops also extend to the enzymes that convert retinol to all-*trans* retinaldehyde as well as other related enzymes (Feng et al., 2010, Strate et al., 2009), including *Ski*, which seem to have cell-type specific roles (Melling et al., 2013, Niederreither and Dolle, 2008).

## Final remarks

The AOP framework holds great promise in supporting chemical risk assessors and regulators. Yet, although conceived over a decade ago, the AOP-Knowledge Base is still lacking a critical mass of fully developed, peer-reviewed and endorsed AOPs. This slow progress reflects the substantial effort and resources required to develop an AOP, such that conception to endorsement can take upwards of three years or longer. Thus, we have recently proposed to use the KER as a discrete unit of knowledge for initial steps of peer review within the wider AOP framework (Svingen et al., 2021). As such, we propose KERs be peer reviewed via journal submission, in isolation from the broader AOP, so that these can be used to more rapidly build robust AOP networks that can be adopted by risk assessors. In line with this proposal, we have here presented a first case study for how a KER can be developed independently of a complete AOP. Our goal is to show, by consideration of one case, how this approach could be used to 1) achieve expert peer review and 2) fast track AOP development. In doing so we are aiming to also expedite the development of the specific AOP to which this KER relates; namely linking inhibition of ALDH1A leading to reduced fertility. Going forward, it will be important to assess how efficient this new method of review is for larger and more complex AOPs than the one we have described here (for example those with multiple MIEs and subsequently many KERs). However, we are hopeful that the large majority of AOPs to be developed this way will profoundly benefit in being made available in a timely manner to chemical risk assessors and regulators than they otherwise would have been.

## Funding

This work was funded by a Tender from the Directorate-General for Environment (DGEM) Belgium, Special Specification No. DGEM/MRBC/CD/20005.

## CRediT authorship contribution statement

**Monica Kam Draskau:** Conceptualization, Methodology, Investigation, Data curation, Writing – original draft, Writing – review & editing, Funding acquisition. **Anne-Sofie Ravn Ballegaard:** Conceptualization, Methodology, Investigation, Data curation, Writing – review & editing, Funding acquisition. **Louise Ramhøj:** Conceptualization, Methodology, Investigation, Data curation, Writing – review & editing, Funding acquisition. **Josephine Bowles:** Conceptualization, Methodology, Investigation, Data curation, Supervision, Writing – review & editing, Funding acquisition, Project administration. **Terje Svingen:** Conceptualization, Methodology, Investigation, Data curation, Writing – original draft, Supervision, Writing – review & editing, Funding acquisition, Project administration. **Cassy M. Spiller:** Conceptualization, Methodology, Investigation, Data curation, Supervision, Writing – review & editing, Funding acquisition, Project administration.

## Declaration of Competing Interest

The authors declare that they have no known competing financial interests or personal relationships that could have appeared to influence the work reported in this paper.
